# Acute obstruction from adult left-sided Bochdalek hernia: a rare surgical emergency

**DOI:** 10.1093/jscr/rjaf352

**Published:** 2025-05-30

**Authors:** Tushar Nagtode, Amol Gupta, Darshana Tote, Swati Deshpande, Jhanwi Khurana, Riddhi Thakor

**Affiliations:** General Surgery, Datta Meghe Institute of Higher Education and Research Deemed to be University, Sawangi, Wardha 442001, Maharashtra, India; General Surgery, Datta Meghe Institute of Higher Education and Research Deemed to be University, Sawangi, Wardha 442001, Maharashtra, India; General Surgery, Datta Meghe Institute of Higher Education and Research Deemed to be University, Sawangi, Wardha 442001, Maharashtra, India; General Surgery, Datta Meghe Institute of Higher Education and Research Deemed to be University, Sawangi, Wardha 442001, Maharashtra, India; General Surgery, Datta Meghe Institute of Higher Education and Research Deemed to be University, Sawangi, Wardha 442001, Maharashtra, India; General Surgery, Datta Meghe Institute of Higher Education and Research Deemed to be University, Sawangi, Wardha 442001, Maharashtra, India

**Keywords:** diaphragmatic hernia, Bochdalek hernia, acute intestinal obstruction

## Abstract

A diaphragmatic hernia is a defect in the diaphragm that allows abdominal contents to enter the chest cavity due to negative intra-thoracic pressure. Diaphragmatic hernias can be acquired (iatrogenic and traumatic), congenital (Morgni hernia, Bochdalek hernia), or hiatal hernia. Bochdalek's hernia (BH) seldom affects adults and usually affects the left side. There are not >100 cases of adult left BH, and the majority of them have no symptoms.

## Introduction

Although adult Bochdalek hernias (BH) are comparatively rare, they are thought to originate during embryogenesis when the backward closure of the diaphragm is failed. About 0.17% to 6% of all diaphragmatic hernias in adults that develop spontaneously are symptomatic BH, making them comparatively rare [1, 2]. There have been about 100 adult cases of BH notified in the records, the majority of which have been left-sided. Usually occurring on the left side, BH involve the stomach, transverse colon, small intestine loops, and fat or omentum [3].

BH is typically diagnosed via Contrast Enhanced Computed Tomography (CECT) because of its increased sensitivity and capacity to identify concurrent congenital abnormalities. CT has 78% sensitivity for left-sided hernias, while for right-sided hernias, it has a 50% sensitivity [4]. Surgical intervention for symptomatic BH involves either thoracic or abdominal approaches to fix the defect, such as open repairs or laparoscopic procedures [5].

## Case presentation

A 44-year male came to the hospital with a complain of pain in the abdomen for 1 day, pain was sudden in onset and gradually progressive. The pain was non-radiating in nature. Breathlessness, haemoptysis, fever, trauma, nausea, vomiting, constipation, or diarrhoea were all denied. On auscultation of the chest, reduced respiratory sounds were observed on the left lower lobe along with gurgling sounds.

**Figure 1:**
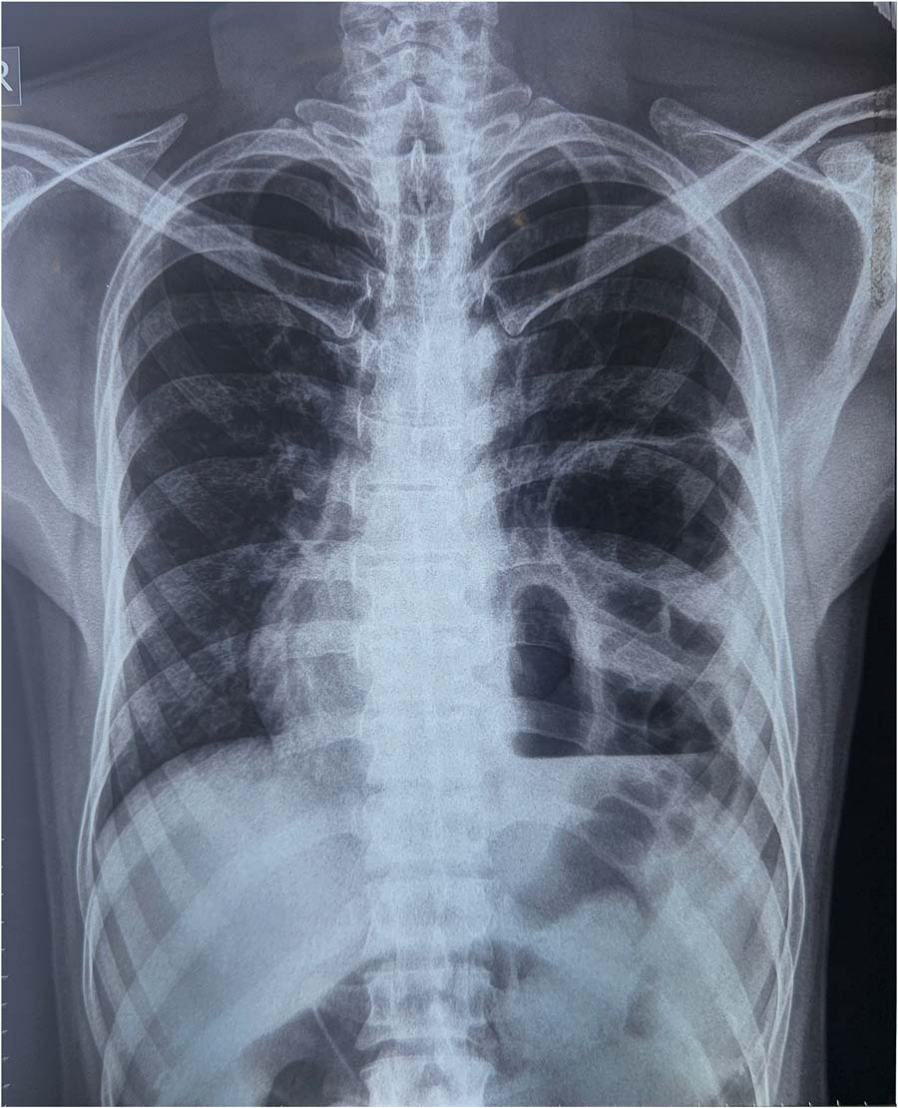
X-ray s/o air-fluid level in left hemithorax.

**Figure 2:**
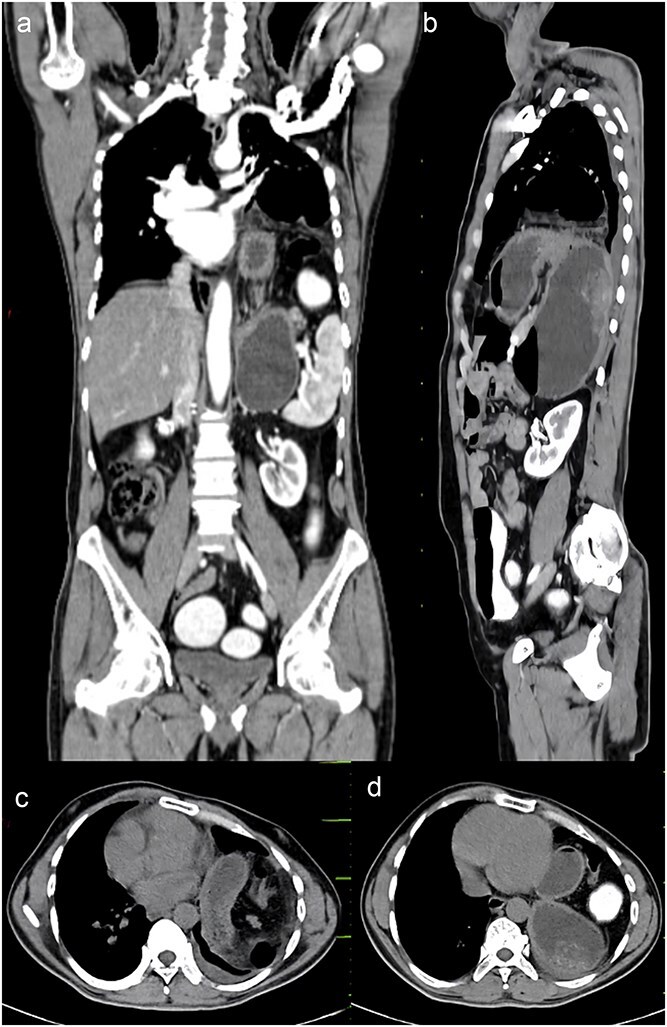
CECT abdomen and pelvis. (a) Sagittal view, (b) coronal view, (c, d) axial view.

A chest x-ray was done, and it showed continuous internal air columns with abdominal gases, with a shifting of cardiac shadow to the contralateral side (Fig. 1). After stabilization of the patient, CECT thorax, abdomen, and pelvis was done which suggests left-sided diaphragmatic hernia with a defect of 28 mm with herniation of splenic flexure, entire left hemithorax. Obstructed loop seen within the herniated content with collapsed exiting loop s/o obstructed diaphragmatic hernia which led to mediastinal shift to right side and collapse of left lung (Fig. 2)*.*

**Figure 3:**
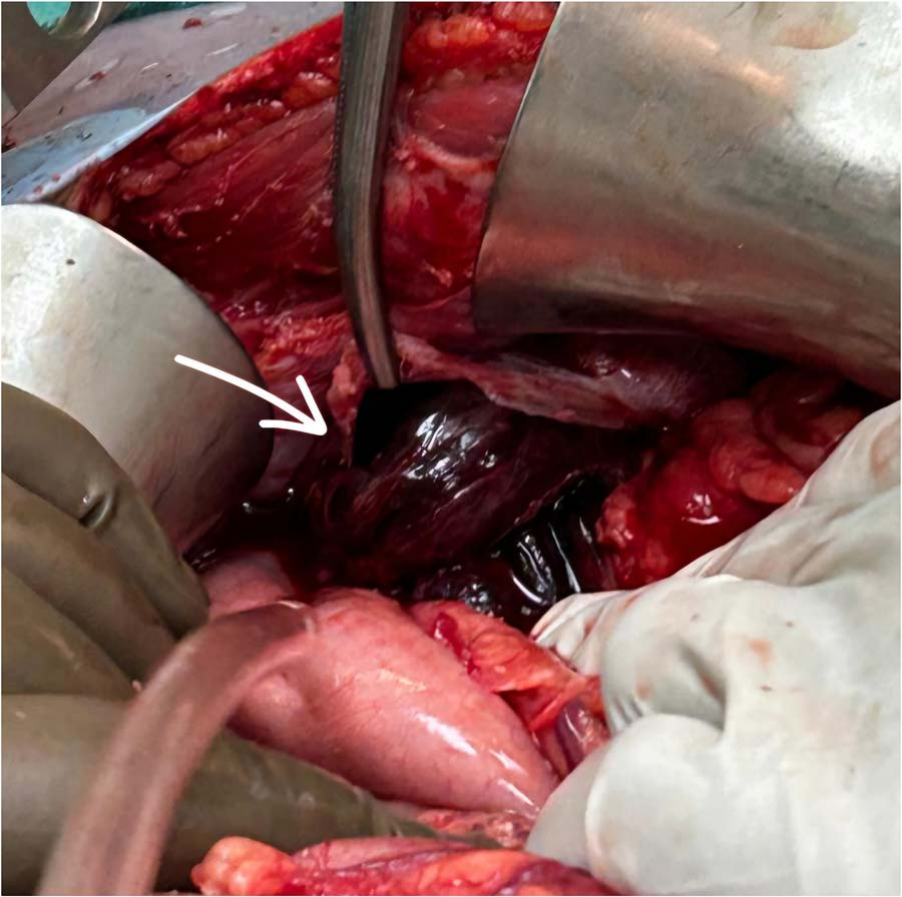
Arrow showing diaphragmatic defect with its contents.

**Figure 4:**
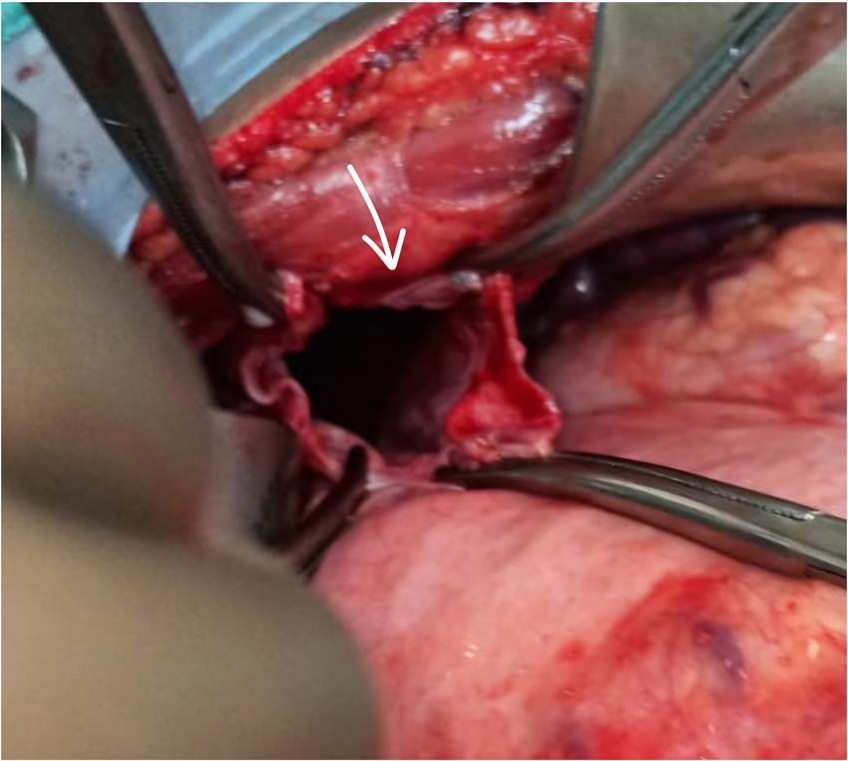
Arrow shows the defect.

**Figure 5:**
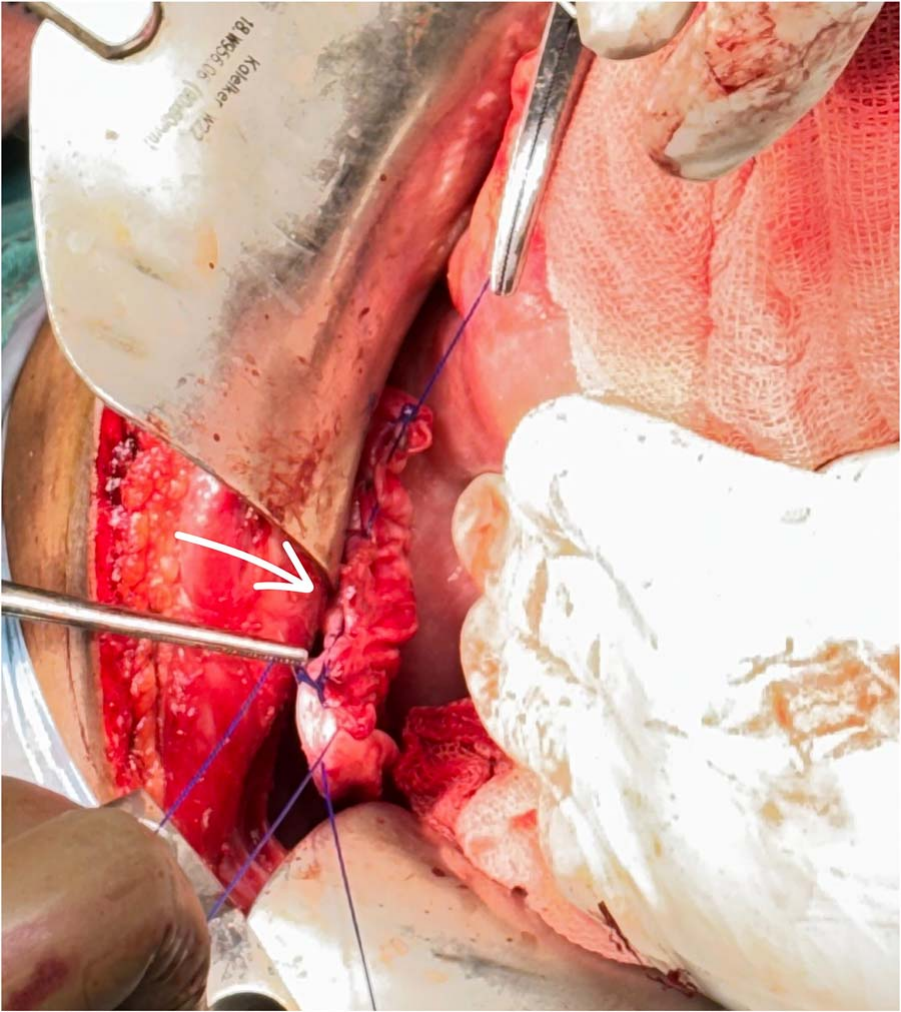
Shows the repaired diaphragmatic hernia defect.

The patient underwent exploratory laparotomy. Left subcostal incision taken, intra-operatively adhesions were noted over the left hemidiaphragm near the defect, and herniation of the splenic flexure of colon along with part of stomach and greater omentum. The part of omentum was found gangrenous (Figs 3 and 4) and rest of the herniating contents was healthy and viable. The herniating content was repositioned in abdomen after adhesiolysis with omentectomy and primary repair of the left diaphragmatic defect using Prolene 1-0 RB (Fig. 5) after refreshening edges of the defect which were later sent for biopsy. The procedure was uneventful. One chest tube was placed in the left hemithorax and one abdominal draining tube was inserted in the subdiaphragmatic space.

Postoperatively the patient was managed in intensive care unit for 2 days with intercostal drain (ICD) and abdominal drain in-situ. On postoperative day (POD) 2 abdominal drain was removed as only 50 ml of serous collection was observed. On POD 5 ICD was removed as no air column movement was observed for 2 days and ICD collection had been decreased to <100 ml/day. Postoperatively, the patient was managed with IV fluids, antibiotics, and analgesics. Once he was hemodynamically and vitally stable, he was discharged on POD 7.

The patient was followed up for 2 months, during which no new respiratory or abdominal complaints were noted.

## Discussion

BH, the most prevalent fetal congenital diaphragmatic hernia, is larger, posterolateral, and is mostly occurring on the left side (75%–90%), arises earlier, and is linked to poor results. In adults, BH is extremely uncommon and typically found by chance [2].

Adults with BH most frequently presented with symptoms of ileus (38%) and chest and/or abdominal pain (66%) [6]. About 65% of BH were left-sided. About 96% of patients had symptoms like discomfort and dyspnoea in their chest and/or abdomen when they first arrived.

A mediastinal shift to the opposite side, cardiac arrest, or a misdiagnosis of pneumothorax could all worsen BH [7]. Chest radiography and thoracic ultrasonography can raise suspicions of BH. Yet, the investigation of choice with the maximum effectiveness for BH is CT. It displays the abdominal structures that are herniating as well as any issues. Certain patients may undergo MR imaging (in cases of late-onset DH and when the diagnosis is still uncertain) [2, 5, 8].

Based on the clinical features and the chest X-ray, a complex left BH was suspected; multislice computed tomography of the chest and abdomen verified the diagnosis. Adult BH can be repaired either by exploratory laparotomy or thoracotomy. For reducing herniating contents into the abdominal cavity diaphragmatic hernias are typically repaired using mesh, or primary closure of defect [9–12]. When adhesions are present around the defect transthoracic approach is beneficial as there is direct vision on the herniating sac [10].

The abdominal approach (laparotomy) is generally favoured in the management of acute diaphragmatic hernias, particularly in traumatic cases. It allows comprehensive inspection and treatment of associated intra-abdominal injuries, which are common in blunt trauma. The approach offers easier reduction of herniated contents and better exposure of the diaphragm from below, making it ideal for early repair and mesh placement if necessary. Additionally, surgeons are typically more familiar with this route in acute trauma settings, and it provides direct access to vital organs like the liver, spleen, and intestines.

While thoracotomy is preferred in chronic or delayed cases due to better access to adhesions, it is more invasive and limits abdominal access. Minimally invasive methods such as laparoscopy are suitable alternatives in stable acute cases, combining diagnostic and therapeutic benefits with reduced morbidity. Overall, the abdominal approach remains the most practical and effective choice in the acute setting.

Following surgery, several patients needed pleural tapping in cases of effusion or mechanical ventilation for breathing assistance. Death or empyema is the most severe respiratory consequence [7].

## Conclusion

Adult BH are uncommon and typically asymptomatic, but to prevent complications, surgery is necessary when they get diagnosed or manifest symptoms. When radiology suggests pneumothorax, BH should be taken into account in the differential diagnosis and treated promptly to prevent problems.
